# Intelligent Navigation of a Magnetic Microrobot with Model-Free Deep Reinforcement Learning in a Real-World Environment

**DOI:** 10.3390/mi15010112

**Published:** 2024-01-09

**Authors:** Amar Salehi, Soleiman Hosseinpour, Nasrollah Tabatabaei, Mahmoud Soltani Firouz, Tingting Yu

**Affiliations:** 1 Department of Mechanical Engineering of Agricultural Machinery, Faculty of Agriculture, University of Tehran, Karaj 31587-77871, Iran; amar.salehi@ut.ac.ir (A.S.); mahsoltani@ut.ac.ir (M.S.F.); 2 Department of Medical Nanotechnology, School of Advanced Technologies in Medicine, Tehran University of Medical Sciences, Tehran 14618-84513, Iran; ntabatabaei@tums.ac.ir; 3 Guangzhou International Campus, South China University of Technology, Guangzhou 511442, China; yu@scut.edu.cn

**Keywords:** autonomous navigation, deep reinforcement learning, intelligent microrobot, model-free control

## Abstract

Microrobotics has opened new horizons for various applications, especially in medicine. However, it also witnessed challenges in achieving maximum optimal performance. One key challenge is the intelligent, autonomous, and precise navigation control of microrobots in fluid environments. The intelligence and autonomy in microrobot control, without the need for prior knowledge of the entire system, can offer significant opportunities in scenarios where their models are unavailable. In this study, two control systems based on model-free deep reinforcement learning were implemented to control the movement of a disk-shaped magnetic microrobot in a real-world environment. The training and results of an off-policy SAC algorithm and an on-policy TRPO algorithm revealed that the microrobot successfully learned the optimal path to reach random target positions. During training, the TRPO exhibited a higher sample efficiency and greater stability. The TRPO and SAC showed 100% and 97.5% success rates in reaching the targets in the evaluation phase, respectively. These findings offer basic insights into achieving intelligent and autonomous navigation control for microrobots to advance their capabilities for various applications.

## 1. Introduction

In recent years, there has been considerable progress in microrobotics applications across many fields, including but not limited to medicine and the food industry [1,2,3]. The significant potential that microrobots carry for use in the general scientific field has gained significant interest in the scientific community. Since the structure of the microrobots is very small, they can be utilized in medicinal fields as they can reach inaccessible areas [4].

Nevertheless, one major challenge in microrobotics is achieving autonomous, intelligent, and precise control and navigation of microrobots toward target positions [5,6]. A microrobot control system aims to manipulate the shape and size of the actuation energy field, focusing on providing navigation for the microrobot toward a desired dynamic behavior. To this end, the microrobots must have the necessary capabilities and tools for sensing the environment, enabling propulsion, and decision-making. While these functionalities are common in macro-robots through the integration of software and hardware tools [7], the tiny size of micro-dimensions will prohibit the integration of hardware tools like sensors, power sources, communication equipment, and more [8]. Therefore, microrobots often come with relatively simple structures. This limitation lays the ground for alternative actuation and navigation approaches. Magnetic microrobots remotely actuated via magnetic fields are approved as a feasible solution to micro-scale propulsion challenges. Due to their safety, especially in medical applications, these microrobots encompass a wide range of navigation and swimming mechanisms in fluidic environments. They also offer other complementary functionalities like cell, drug, and gene delivery [9], biopsy [10], and disease diagnosis [11].

However, the efficient and autonomous motion control and navigation of these magnetic microrobots in fluidic environments require intelligent, precise, and optimal methods. Traditional methods for microrobots’ control and navigation typically employ approximate and experimental methods, which ultimately need complex dynamic modeling of the whole system including microrobot dynamics, environmental conditions, and actuation systems [12]. This limitation restricts the flexibility and adaptability of these approaches for implementation in various control situations, especially within complex and unpredictable environments.

Recent developments in artificial intelligence (AI) and deep learning (DL) technologies [13] have introduced reinforcement learning (RL) algorithms as a possible solution to the problem of microrobot motion control [14]. The combination of RL with DL called Deep RL (DRL), is most useful in complex decision-making tasks where effectively learning complex behaviors and features is essential [15]. The main advantage of model-free DRL algorithms relies on the fact that they do not need a predefined model for the prediction of various environmental states. This, therefore, simplifies their implementation, particularly in unpredictable environments.

Considering the promising success demonstrated by RL algorithms in a wide range of robotic control problems [16,17] as well as the inherent complexity of environments in which microrobots operate, recent studies have focused on the use of RL algorithms in microrobot control and navigation. For instance, Q-learning as a value-based RL algorithm has been employed to learn appropriate policies for movement in steady flow [18], optimization of the propulsion policies for three-sphere microswimmers [19], motion control in simulated blood vessels environments [20], and support microrobot real-time path planning in navigation [21]. However, Q-learning not only lacks performance efficiency in systems with continuous action spaces [22] but also exhibits a high degree of sample inefficiency [23,24]. This limitation results in multiple challenges in practical, real-world scenarios that typically require continuous action spaces and sample efficiency.

The actor-critic approach is introduced to overcome these limitations. Recent studies have reported that microrobots can be controlled and guided properly within complex environments using the actor-critic strategy. So far, this approach has been applied for the gait learning in microrobots to swim into predefined directions [25], guiding microrobots to target areas under diverse environmental conditions [26], and learning how to use hydrodynamic forces to move in a controlled direction [27]. Nevertheless, most of these studies have been dedicated to deploying RL approaches only under very simple settings and assumptions in computational modeling and simulation.

Soft actor-critic (SAC) [28], a prominent actor-critic approach, has recently shown promising results in the control of microrobots in real-world environments. For example, Behrens and Ruder (2022) applied this algorithm to control the movement of an intelligent helical magnetic microrobot [29]. In their study, a rotating electromagnetic system was used to obtain a nonlinear actuation. The optimal swimming behaviors were acquired autonomously in the fluid environments; thus, the need for prior knowledge of the environmental conditions was eliminated. In this way, the development time and resource utilization required for designing the system was minimized accordingly. The RL agent’s objective was to learn an action policy that maximizes overall reward for guiding the microrobot in a circular workspace toward the target position. The results showed that the SAC algorithm successfully learned a nearly optimal policy for the circular motion of the microrobot around a fixed point within the fluid environment [29]. In a separate study, the SAC algorithm was used to control a soft magnetic microrobot to reach a target point for simulating drug delivery and then float within the fluid for simulating drug release [30].

Despite the acceptable results of the SAC in controlling microrobots in real-world environments, one limitation of the SAC is training instability, in which one can observe a huge degradation in performance during the training process. This limitation seems to be responsible for non-optimal behaviors, such as the negative backward movement of the microrobot reported by Behrens and Ruder (2022) [29]. To address this issue, the Trust Region Policy Optimization (TRPO) algorithm [31] is a feasible solution because it utilizes the concept of trust region to prevent large updates during each iteration. To the best of our knowledge, none of the previous studies employed this algorithm for the position control of microrobots.

In the present study, both the TRPO and SAC algorithms were independently employed to control a single magnetic disk-shaped microrobot within the physical system, and their respective results were subsequently compared. For this purpose, a magnetic actuation system with two rotating permanent magnets and a uniaxial Helmholtz coil was assembled. RL algorithms enable the microrobot to efficiently navigate from an initial position to an arbitrary goal position in the shortest path. The microrobot’s task was to determine the optimal or near-optimal policy for reaching the target positions on an air-water interface. The primary contribution of this paper is to provide a framework for implementing two different DRL algorithms to enable microrobot operations in such circumstances. In order to compare the results, the evaluations were performed for each algorithm. In this research, we did not involve any analysis of the magnetic field modeling generated by the actuation system or the dynamic modeling of microrobot behavior within the fluid environment. The hypothesis of this study revolves around the development of an RL-based control system that does not need to develop a specialized model of the whole physical system.

## 2. Materials and Methods

### 2.1. The Microrobot and Magnetic Actuation System

**Figure 1:**
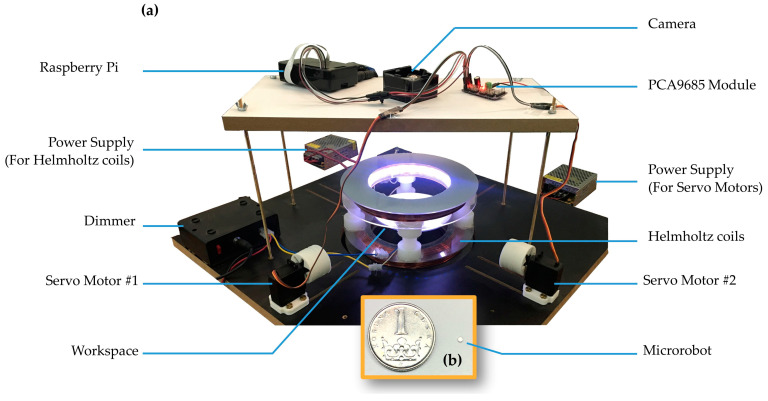
The microrobot and magnetic actuation system: (**a**) the actuation system consists of a pair of Helmholtz coils and two rotating permanent magnets, (**b**) a photograph of the microrobot next to a 20 mm coin.

In this study, a magnetic actuation system was fabricated inspired by the work of Yousefi and Pishkenari (2021) [32] to evaluate the effectiveness of the RL approach in controlling a disk-shaped magnetic microrobot (Figure 1). The microrobot was made of neodymium alloy (NdFeB) grade N45 with three protective layers of nickel and cobalt and axial magnetization (Webcraft Co., Gottmadingen, Germany). The microrobot had dimensions of a 750-micrometer radius and a 500-micrometer thickness, as shown in Figure 1b. The magnetic actuation system comprises two rotating permanent magnets and a pair of uniaxial Helmholtz coils. Both of the permanent magnets are cube-shaped with dimensions of 20 × 20 × 20 mm^3^ and made of N42 grade neodymium alloy (NdFeB) with three protective layers of nickel and cobalt. Two MG996R servo motors (Tower Pro Pte Ltd., Singapore, Singapore) with a working angle range of 180° were used to rotate the magnets. Since the magnets have two positive and negative poles in the axial direction and generate a magnetic field flux density of 1.32 tesla, altering their angle will change the magnetic field and its gradient within the workspace. Consequently, this leads to changes in the position of the microrobot that floats on the surface of the liquid along the XY plane with two degrees of freedom. Each magnet has one rotational degree of freedom; therefore, using two magnets establishes a fully-actuated control system. The servo motors and the permanent magnets were positioned 17 cm away from the center of the workspace. Figure 1a displays the fabricated magnetic actuation system.

Additionally, the rotation of the servo motors was controlled using a 3B V1.2 Raspberry Pi board (Raspberry Pi Foundation, Cambridge, UK). The servo motors were connected to the Raspberry Pi via a 16-channel 12-bit PWM driver module featuring a PCA9685 chip (Adafruit Industries, New York, NY, USA). This driver module was connected to a 5-volt power supply with a 5-amp current on one side and to the Raspberry Pi board through a GPIO connector and an I2C communication protocol on the other. The 2D workspace restricted the motion of the microrobot within the XY plane, so a uniaxial Helmholtz coil was used to generate a uniform and strong magnetic field. This field served to align the microrobot and, therefore, orient its magnetic moment perpendicular to the workspace, specifically in the Z-direction. The coils have an average diameter of 15 cm with 166 turns, capable of generating a magnetic field of 9.6 mT strength. The workspace consisted of a petri dish with a 6 cm diameter centrally placed within the coils. Deionized water was also used to prevent any formation of air bubbles in the petri dish during the learning process.

### 2.2. Image Processing for Microrobot Detection

Image processing was carried out to detect and determine the position of the microrobot within the XY plane. This involved using a USB camera with a resolution of 1920 × 1080, operating at 30 frames per second. A 15-watt LED strip and a 3000-watt dimmer were employed to adjust the light intensity (Figure 1) to ensure proper lighting conditions for effective object detection. The image processing was carried out using the OpenCV library in Python version 3.10.9. In the first stage, the most recent frame obtained from the camera was cropped and then processed through color image processing. In this phase, the image was converted to grayscale to simplify further operations. Then, the grayscale image was converted to a binary image by employing adaptive thresholding. In the next step, two fundamental morphology operations, namely Erosion and Dilation, were applied to correct and refine the binary image. This was followed by finding contours outlining the boundaries of connected objects in the binary image. Eventually, the largest contour, which is the most prominent object in the image, was utilized to accurately detect the microrobot and its position.

### 2.3. RL Approach

The operation of RL involves interaction with the environment through a series of time steps to acquire the optimal policy that maximizes the cumulative rewards. RL typically requires a basic mathematical framework to understand and solve the associated problems. The Markov Decision Process (MDP) offers a structured framework for representing such decision problems. A MDP can be defined as $M=\langle S,A,P,R,\gamma \rangle$, where S is the state space, A is the action space, $P(s_{t+1}|s_{t},a_{t})$ is the transition probability distribution, $R(s_{t},a_{t})$ is the reward function, and γ is the discount factor [33]. An agent in its initial state s_1_ starts from a fixed distribution $P\left(s_{1}\right)$, takes an action $a_{t}\in A$ of one state $s_{t}\in S$, and transitions to the next state $s_{t+1}∼P(\cdot |s_{t},a_{t})$ at each time step *t*. After each action, the agent receives a reward $r_{t}=R(s_{t},a_{t})$. In a standard RL algorithm, the primary objective of the agent is to learn the optimal policy $\pi (a_{t}|s_{t})$ to maximize the cumulative reward (Equation (1)) [33].

(1) $$\sum_{t=1}^{T}E_{(s_{t},a_{t})∼\rho_{\pi }}\left\lceil r_{t}\right\rceil$$

where *T* is the total number of time steps and $\rho_{\pi }$ is discounted state-action visitations.

Numerous algorithms have been developed to align with this framework. The MDP problem in this study is structured as follows: in each state, the microrobot can select an action. If the action falls outside a predefined range, a minor penalty is applied. Once a valid action is determined, the microrobot transitions from its current state to a new one. The rewards for the microrobot depend on whether it reaches the predefined goals, where it receives a bonus reward. Reaching locations other than the desired goal results in a slight penalty (negative reward).

In Section 2.3.1, the characteristics of the environment and the reward function are first defined. Then, the SAC and TRPO algorithms are discussed in Section 2.3.2.

#### 2.3.1. The Environment Characteristics and Reward Function

To implement a DRL algorithm, it is necessary to specify the state space, action space, and rewards for the agent within the system. In this study, the control objective was to change the angle of permanent magnets to achieve the desired dynamic behavior of the microrobot. The RL algorithms were implemented in Python using the Stable-Baselines3 (SB3) library to control the position of the microrobot floating on the water surface. Since the SB3 library requires an environment for RL algorithm implementation, a custom environment was developed using a standard Gymnasium API provided by OpenAI (OpenAI, San Francisco, CA, USA). In addition, an episodic problem with continuous action and state spaces was formulated for the control problem. The microrobot position (x and y), magnets angles (angle1 and angle2), and random positions in the workspace (x_t_ and y_t_) were defined as state space, action space, and target position in the custom environment, respectively. The workspace was defined as a gridworld of size of 134 × 134 pixels with 17 pixel margins. During each episode, one target position was randomly selected from a set of five predefined positions. Furthermore, states, target positions, and actions were normalized to ensure data comparability. So, the action values generated by RL algorithms were constrained within the range of (−1, 1). These values were then converted into angles of the permanent magnets between 0 to 180°. However, to prevent violent and sudden movements of the microrobot within the workspace, the action values were then rescaled to a range between (−10, +10) degrees rather than accessing the full range of servo motor angles (0, 180) directly. In other words, after the RL algorithm calculated two numbers from the range (−1, +1) as RL actions at each time step, they were then rescaled, as described in Equation (2), and then used independently as new actuation commands (*cmd_i_*) for both servo motors. At the beginning of each episode, the servo motor angles were reset to *cmd*_0_ = 90°.

(2) $${cmd}_{i}={(a}_{t}\times 10)+{cmd}_{i-1}$$

where *i* is the time step and a*_t_* is the action calculated by RL algorithms.

This configuration resulted in a smooth movement of the microrobot in the workspace. Also, given the limitations of servo motor angles and to train the agent to avoid sending actuation commands outside the 0 to 180° range, a control penalty of −0.5 was defined when the angle of each servo motor was out of the range.

Each episode was structured to terminate either when the microrobot reached the target position or after a maximum of 100 time steps, at which point, the next episode would start. After choosing an action by the agent, the camera and image processing determined the microrobot position. Then, an observation reward function was calculated based on the Euclidean distance (*d*) between the microrobot and the target position, according to Equation (3):

(3) $$Observation\;Reward=\left\{\begin{array}{ll} +40, & d<2\\ -1, & microrobot\;hits\;the\;border\\ r_{c}\times d, & elsewhere \end{array}\right.$$

where *r_c_* = −0.007 is a reward constant used to normalize the penalty.

Here, if the Euclidean distance was less than 2 pixels, the microrobot was rewarded with a large bonus. This reward was designed to encourage the agent to reach the target promptly. Furthermore, a constant penalty of −1 was introduced to prevent the microrobot from hitting the boundaries of the workspace. Lastly, a dynamic penalty ranging between −1 and 0 was applied for the agent based on the Euclidean distance. The final reward was determined as the sum of the control reward and the observation reward. As a result, the reward obtained for each episode is bounded within the range of (−190, +40) from reaching the goal within a single time step (+40) through to continuous collision with the workspace boundaries and selection of both servo motors angles outside the range at all time steps.

#### 2.3.2. Implementation of the SAC and TRPO Algorithms

SAC is a Deep RL (DRL) actor-critic algorithm that uses an off-policy approach, which distinguishes between the data collection policy and the agent policy during the learning process through an entropy regularization framework [34]. This algorithm uses a replay buffer to store past experiences and reuse them, so it reduces the need for the agent to collect new samples all the time in each iteration. SAC employs three neural networks that not only compute the action predictions for the current state but also generate a temporal-difference error signal for every time step. These three networks are the actor network to approximate the policy, the value network to approximate the state value, and finally, the critic network to approximate the Q-value [35].

The neural network used in the SAC algorithm comprises ten inputs as follows: four inputs representing the four most recent observations of the microrobot’s x position, four inputs representing the four most recent observations of the microrobot’s y position, and two inputs representing the target’s x_t_ and y_t_ positions. The history of the last four observations was included in the neural network due to a time delay between action and observation within the physical system. The outputs of the neural network were also sent to the corresponding servo motors as the angles of the permanent magnets according to Equation (2).

**Figure 2:**
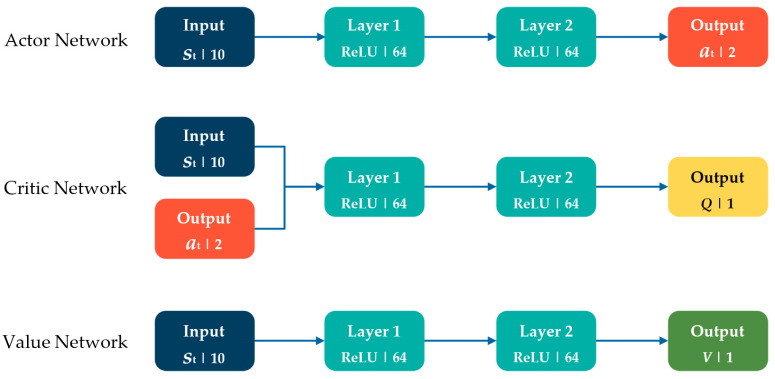
The neural network structure of the SAC algorithm.

The neural network structure of the SAC algorithm is illustrated in Figure 2. The input for the actor network was the current position of the microrobot within the environment. Two layers of the fully connected neural network of 64 nodes were used to connect the input to the output layer. A Rectified Linear Unit (ReLU) was also employed as the activation function. On the other hand, the current state and agent’s action were provided as inputs to the critic network. The output of this critic network represents the Q-value. Eventually, the value network estimated the current state value. These two networks also employed two layers of fully connected neural networks to process the inputs. The Q-value and the current state value were both activated through a linear activation function.

**Figure 3:**
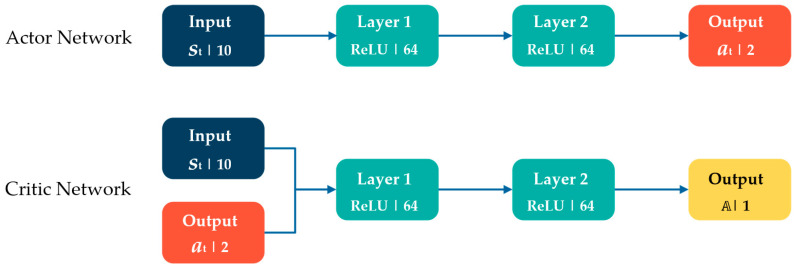
The neural network structure of the TRPO algorithm.

On the other hand, TRPO is a DRL actor-critic algorithm that uses an on-policy approach to employ the same policy for both exploration and exploitation. One of the primary contributions of this algorithm is that it uses a trust region [36] to restrict the update size during training, which ensures monotonic improvement [31]. TRPO employs two neural networks (actor and critic networks) to collect samples and refine the current policy. Similar to the SAC algorithm, the input layer in the actor network is observations from the environment, while the output layer provides the actions. Additionally, the input layer of the critic network consists of the observations from the environment. As an additional input, it also receives actions taken by the agent. The output of the citric network is an advantage function (A) which is employed to make the training updates. The network configuration of the TRPO was similar to the SAC algorithm in terms of layers and activation functions to ensure comparable results, as shown in Figure 3.

**Figure 4:**
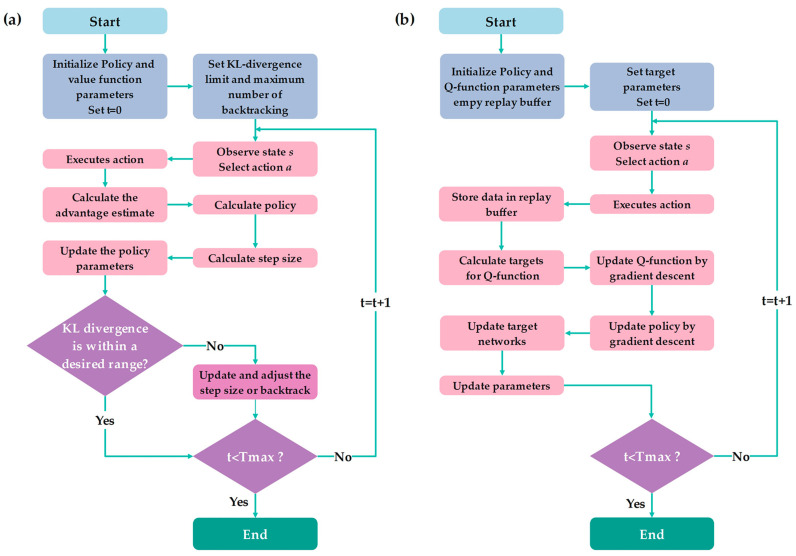
The flow charts of (**a**) the TRPO and (**b**) the SAC.

Figure 4 shows flowcharts of the operation process of the TRPO and SAC algorithms. As shown in Figure 4a, in the TRPO algorithm, there is an initialization of policy and value functions’ parameters. Then, it performs an action *a* selected based on the policy. After an action is executed, the algorithm calculates the advantage estimate based on the current value function and estimates the policy gradient to update parameters. The TRPO is different from SAC in that it provides stability through the regulation of step sizes and Kullback-Leibler (KL) divergence monitoring. The algorithm improves its policy every iteration to provide maximum cumulative rewards. This process continues until it meets the desired policy divergence. On the other hand, in the SAC algorithm, the Actor generates actions through a decision function, while the Critic makes predictions based on the actions taken by the Actor. After initializing the policy parameters, the system obtains the state *s* from the environment. The Actor randomly executes an action *a*, and the Critic evaluates this action based on the *s* and *a*. The data, including *s*, *a*, the next state *s*ʹ, reward *r*, and done signal *d*, are stored in the replay buffer. The Actor leverages the critic evaluation to maximize entropy. The Critic compares its evaluation with the reward value from the environmental feedback. Finally, back-propagation is employed to update the network parameters, as illustrated in Figure 4b.

**Figure 5:**
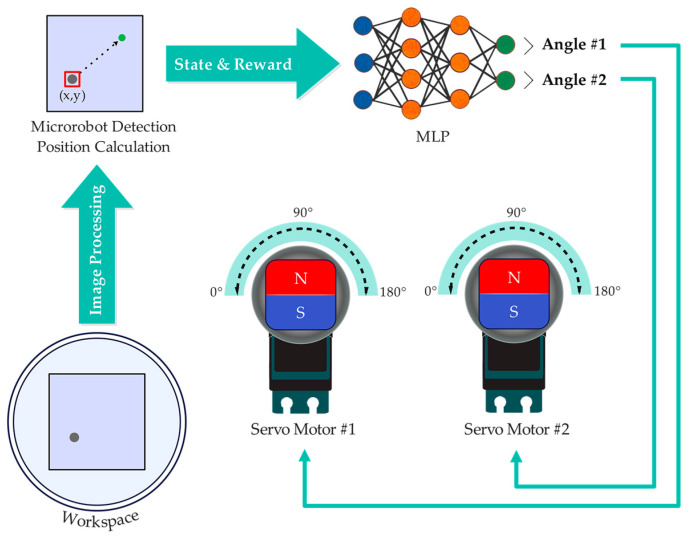
Diagram of receiving inputs from the overhead camera and sending outputs (angle #1 and angle #2) to the servo motors for rotating the magnets.

The SAC and TRPO algorithms were implemented on an Acer Aspire 3 A315-57G-77K6 laptop (Acer Inc., New Taipei City, Taiwan) with an Intel i7 1065G7 CPU and 8 GB RAM (Intel Corporation, Santa Clara, CA, USA). This laptop received the microrobot’s position via the overhead camera and image processing, then transmitted the permanent magnet angles to the Raspberry Pi through a TCP/IP communication protocol using a local Wi-Fi network and the socket library in Python. After receiving the data, the Raspberry Pi sent it to the PCA9685 driver module through the I2C communication protocol, and this module transmitted the obtained angles to the corresponding servo motors (Figure 5).

The microrobot was trained with each algorithm independently for a maximum of 100,000 time steps or 20 h of agent-experience time and about 45 h of total real-time. The training results of both SAC and TRPO algorithms were monitored using the TensorboardX visualization tool and compared based on criteria including the mean episode reward and the mean episode length. The hyperparameters employed for both algorithms are detailed in Table 1. Considering that the input in this study consists of a state vector, a multi-layer perceptron policy (MLP) was used for both algorithms. Additionally, it is important to highlight that the buffer size is a special hyperparameter used in the SAC algorithm, and the generalized advantage estimation (GAE) coefficient is a unique hyperparameter employed in the TRPO algorithm. The obtained reward is an intrinsic aspect of the agent’s performance. After the training process, the SAC and TRPO agents were evaluated for 40 episodes. The evaluation tests were conducted in untrained scenarios, with the microrobot’s initial positions and target positions differing from those used during the training phase.

## 3. Results

**Figure 6:**
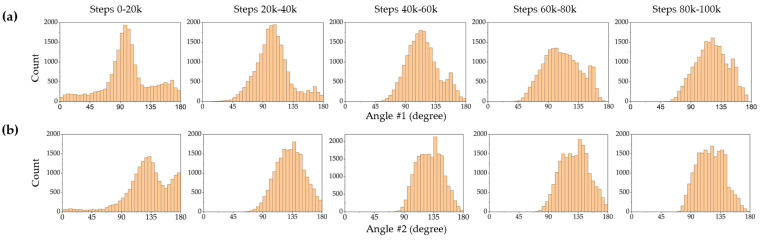
The SAC network outputs (actions) distribution diagram at various time steps: (**a**) angles of the first permanent magnet, (**b**) angles of the second permanent magnet.

**Figure 7:**
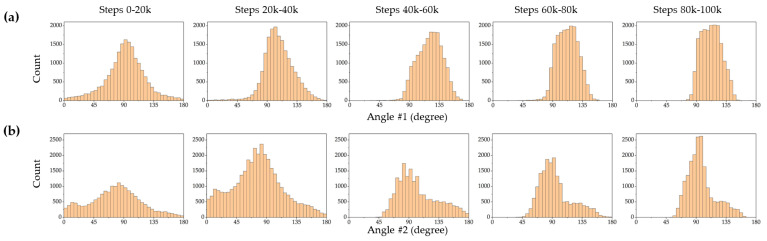
The TRPO network outputs (actions) distribution diagram at various time steps: (**a**) angles of the first permanent magnet, (**b**) angles of the second permanent magnet.

In this study, two different model-free DRL algorithms, TRPO (an on-policy algorithm) and SAC (an off-policy algorithm) were employed to find optimal policies for closed-loop control of a magnetic microrobot on a fluid surface with no prior knowledge of the system dynamic. Frequency distribution diagrams of actions were plotted to gain insights into the SAC and TRPO algorithms’ behavior and performance. These diagrams provide a visual representation of how frequently certain actions are chosen during the learning process. Figure 6 and Figure 7 depict frequency distribution diagrams of the SAC and TRPO algorithm action outputs, respectively, displaying angle 1 (the angle of the first magnet rotated by the servo motor #1) and angle 2 (the angle of the second magnet rotated by the servo motor #2). These histograms are categorized into five intervals: 0–20 k time steps, 20 k–40 k time steps, 40 k–60 k time steps, 60 k–80 k time steps, and 80 k–100 k time steps. These five distinct intervals allow us to analyze the evolution of action distributions over different training phases. Over the initial 20 k time steps, the action distributions are concentrated around a peak, except for angle #2 in the SAC (Figure 6b), which displays two sharp peaks. This indicates that the SAC network tended to rotate the second magnet more frequently with angles exceeding 90°, while the TRPO network tried to rotate both magnets similarly, exploring the entire action space and identifying patterns related to state changes. In this phase, the microrobot explored all possible states within the workspace and exhibited the lowest success rate in reaching the target positions. In the subsequent time steps, from 20 k to 60 k, both networks explore a wider range of angles. Between 60 k and 100 k time steps, the action distributions become more skewed, with some histograms shifted on one side. This implies that both networks discovered near-optimal policies and learned to favor certain angles over others. Notably, at the final 20 k time steps, the action distributions became more uniform and flat (except for angle #2 in the TRPO, as shown in Figure 7b), with some histograms showing almost no peaks. In this phase, the microrobot exhibited more precise and successful navigation toward the target positions. It is important to highlight that in the initial steps, the agents generated non-valid angles (less than zero degrees and more than 180°), which is a natural part of the exploration process for the algorithms. However, as learning progressed, both algorithms effectively learned to avoid selecting invalid angles. In the last 20 k time steps, particularly in the case of the TRPO, they even avoided choosing angles between 170 to 180°.

**Figure 8:**
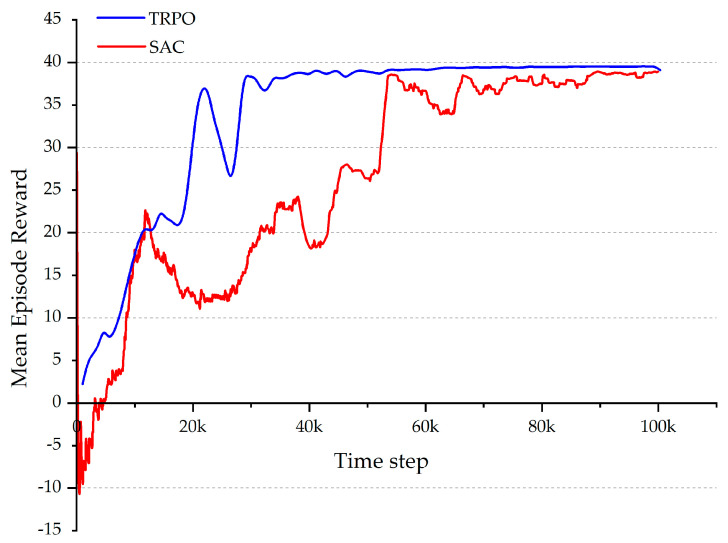
The mean episode reward during training for the TRPO (blue) and the SAC (red).

**Figure 9:**
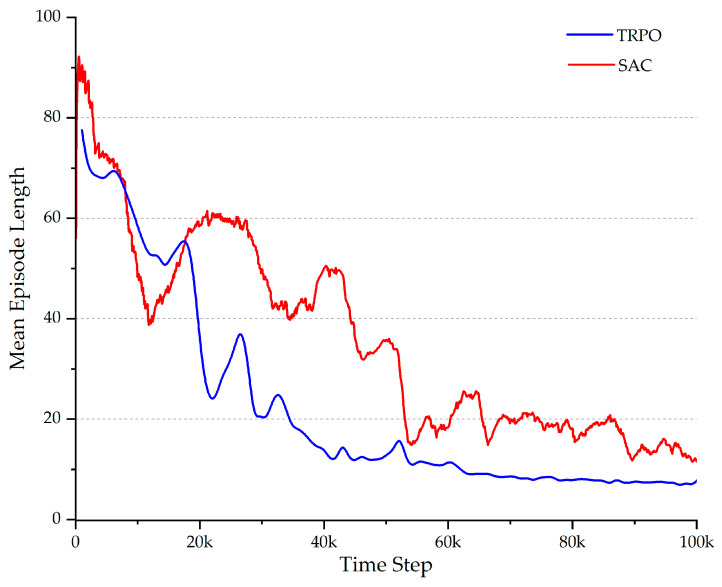
The mean episode length during training for the TRPO (blue) and the SAC (red).

On the other hand, the mean episode length and the mean episode reward over the time steps are important criteria for evaluating the RL effectiveness. Based on the configuration of the system, shorter episode lengths and higher episode rewards are expected to result in better learning efficiency. As illustrated in Figure 8, the TRPO network was able to attain a convergence to an optimal reward at an early stage. In contrast, the SAC algorithm exhibited negative rewards within the first 5000 time steps and then followed by unstable reward values. Figure 8 and Figure 9 illustrate that in the initial episodes for both algorithms, the mean episode length is long, while the mean reward remains low. This is typical for the learning process, where the agent explores the environment and seeks to reach the targets through random actions.

Furthermore, a discernible trend of decreasing episode length and increasing episode reward can be observed with the advancement of training, suggesting that the microrobot’s performance improves with experience. After approximately 30 k time steps, the TRPO succeeded in the task of finding a stable and optimal policy with the maximum possible reward. The SAC, on the other hand, not only took more time steps to reach the maximum possible reward but also exhibited more fluctuations during the training. This indicates that the SAC network does not exhibit the same degree of consistency as the TRPO network. As shown in Figure 9, the mean episode length of the TRPO mostly remained considerably lower than that of the SAC over time, showing that the TRPO reached the target positions more rapidly with less number of time steps.

In the final time steps, both episode length and reward seem to stabilize toward relatively constant values. At this point, the agent was converging to an almost optimal policy with a high degree of robustness, especially in the case of the TRPO. These results suggest that the TRPO is more stable during the learning process compared to the SAC since there are fewer fluctuations in the reward curve throughout training. This could be caused by the SAC’s stochasticity and regularization, which can cause learning curve fluctuations. Another factor attributable to the observed fluctuations on the learning curve could be the off-policy nature of the SAC, which might make it more sensitive to the quality and diversity of the experience data it collects.

**Figure 10:**
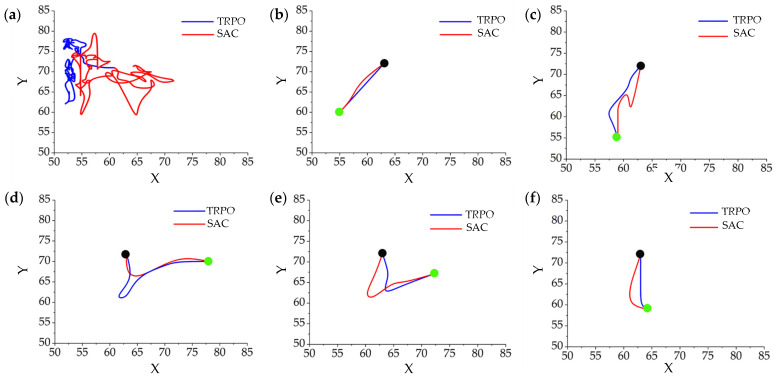
Representative trajectories from an initial position (black circle) at different learning episodes for the microrobot, using SAC and TRPO: (**a**) the first episode with 100 time steps, (**b**–**f**) the final episodes, each with different targets (green circles). (The graphs’ units are represented in pixels).

Figure 10 depicts the algorithms’ performance in the initial episode and final episodes. As anticipated with RL algorithms, in the initial episode, the agent interacted with the environment by generating random actions to explore it. As illustrated in Figure 10a, the first episode had the longest duration (i.e., 100 time steps) and the lowest reward for both SAC and TRPO algorithms. The microrobot learned to find the optimal policy for reaching the target points during the learning process. Figure 10b–f illustrates the trajectories of the microrobot to reach five different targets (green circles) in the final episodes, using both the SAC and TRPO algorithms. This figure shows that both algorithms discover near-optimal policies to reach different targets solely through trial and error and interaction with the environment.

Furthermore, it is worth noting that after the entire learning experience was completed, the TRPO managed to achieve higher success rates in completing episodes compared to the SAC. The results revealed that the TRPO performed 7797 episodes in the learning phase and successfully reached the target positions in 7633 episodes (97.9%). In contrast, the SAC completed 3968 episodes, with 3541 of them successfully reaching the target positions (89%). It appears that the TRPO was able to make more efficient use of the episodes it experienced. This observation is also supported by the success rates of each agent in the initial time steps. In the first 30k time steps especially, the TRPO algorithm saw the microrobot failing to reach the target positions only 21% of the time, whereas the SAC algorithm exhibited a failure rate of 52%. Therefore, the results imply that the TRPO is more sample-efficient than the SAC, which means it requires less sample to reach an optimal policy. Having a sample efficient agent is critical for implementing RL in microrobotics, especially when training with physical systems.

The performance of each agent was then tested under scenarios in which they had not been trained, such as different initial positions for the microrobot and different target positions, to evaluate the generalization of the algorithms. From the mean reward and accuracy results, it is observed that both SAC and TRPO methods can accomplish the goal-reaching task effectively. Additionally, there is not much difference between the SAC and TRPO in achieving the optimal policy, as shown in Table 2. However, the SAC has an immensely lower standard deviation than the TRPO. Standard deviation measures the spread or variability in rewards. This could be due to the SAC’s ability to explore and exploit the policy more effectively. These results provide insights into agents’ generalization and adaptation capabilities, as well as their robustness to uncertainty and noise.

**Figure 11:**
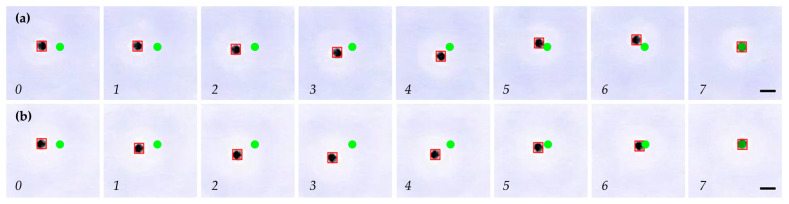
Time-lapse images of the trajectory of the microrobot (black circle with red square border) for reaching the target point (green circle) in 7 time steps in the physical environment during the evaluation process of (**a**) SAC algorithm, and (**b**) TRPO algorithm. (Scale bar: 2.5 mm).

Figure 11 illustrates a time-lapse image of the magnetic microrobot’s trajectory during one of the episodes of the evaluation process of the SAC (Figure 11a) and TRPO (Figure 11b) algorithms in the physical environment. Each episode consists of seven time steps. As is evident from the figure, both algorithms successfully applied their experience gained during the training to navigate the microrobot to the target position. It is important to highlight that to assess the algorithms’ performance in scenarios where they had not been trained, the microrobot’s initial position and the target positions during the evaluation differed from those in the training phase.

## 4. Discussion

The SAC and TRPO algorithms were individually implemented within 100,000 time steps, and their performances were compared. Both algorithms were able to discover and learn optimal policies for their control task. The implementation outcomes of both algorithms demonstrate that the microrobot can effectively learn to navigate through the environment. Compared to other studies focused on closed-loop control of microrobots [22,37], which often rely on complex models, the DRL-based control systems presented in this study require no prior knowledge of microrobot dynamics, the environment, or the actuation system to perform navigation tasks. This adaptability is one of the key advantages of the control systems presented in this study. Additionally, they hold the potential to surpass classical control systems because DRL agents learned from the actual physical behaviors of the system and leveraged deep neural networks to model any observed nonlinearities inherent to the microrobotic systems. Therefore, the current paper can serve as a foundational framework for leveraging DRL to enable microrobots to operate in environments where their models are unavailable.

It was also demonstrated that the SAC algorithm requires a limited number of samples to discover the optimal policy. This high efficiency was also reported by Behrens and Ruder (2022) [29] and Cai et al. (2022) [30], who utilized the SAC algorithm for microrobotic control tasks within physical systems. Nonetheless, in our specific experimental setup, the TRPO algorithm proved to be more sample-efficient than the SAC under the same conditions. One major reason behind the difference in sample efficiency may be found in the design principles of the two algorithms. SAC is known to make use of off-policy learning so that it can efficiently use data collected throughout training. The TRPO, on the other hand, builds on inherently conservative trust region methods. This conservativeness can hence lead to even better sample efficiency in some scenarios.

During the training, the SAC algorithm exhibited frequent fluctuations and huge drops in performance, while the TRPO algorithm maintained greater stability and demonstrated relatively fewer fluctuations. SAC uses stochastic policies that add randomness to actions by including an entropy term. Although this may improve exploration, it also can result in fluctuations in performance. The TRPO, on the other hand, optimizes deterministic policies, which generally exhibit less variability in behavior.

Considering factors such as sample efficiency, learning stability, and no need for prior knowledge about the environmental dynamics or the actuation systems, we encourage the utilization of the TRPO algorithm for microrobotic applications in physical systems, such as drug delivery. Future research should focus on varying environmental conditions encompassing fluid dynamics, obstacle avoidance, path tracking, and navigation in complex environments. Extending the control of microrobots into three dimensions promises more realistic applications. Moreover, exploring alternative microrobots like helical microrobots, distinct actuation methods such as ultrasound, and diverse imaging systems such as MRI should be considered for future studies. This research also has the potential for extension into multi-agent systems, where the learning capabilities can facilitate microrobots in collaborative tasks.

## 5. Conclusions

In this research, two DRL-based closed-loop control systems were separately implemented for the motion and navigation control of a magnetic microrobot on a fluid surface in a real-world environment. To achieve this objective, we implemented an off-policy algorithm, SAC, and an on-policy algorithm, TRPO. Experiments were performed to demonstrate the feasibility of navigating the microrobot from its initial position to random target positions on the liquid surface without any prior knowledge of the microrobot’s dynamics, the actuation system, or the environment. The microrobot was actuated by a magnetic actuation system consisting of two rotating permanent magnets and a uniaxial Helmholtz coil. Both algorithms trained the microrobot under identical conditions, using the same inputs and outputs, as well as the same hyperparameters, for a total of 100,000 time steps. The primary feedback for both control systems was established through a defined reward function. The results of the training and evaluation phases indicate that both the SAC and TRPO algorithms successfully discovered and learned the optimal path for the microrobot to reach random target positions with acceptable accuracy and a limited number of time steps. The microrobot in this study effectively learned magnetic actuation policies from system state inputs without any prior knowledge or modeling of the microrobot’s dynamics, the magnetic actuation system, or the environment. The findings proved that the TRPO algorithm exhibits higher sample efficiency and greater stability during training in comparison to the SAC algorithm. These characteristics are crucial for microrobotic tasks in real-world physical systems. The DRL-based control systems in this study can decrease the time and resources required for the development of high-performance microrobotic systems by learning control policies without requiring highly specialized models.

## Figures and Tables

**Table 1:** The hyperparameters for both algorithms.

Hyperparameters	SAC	TRPO
Time steps	100,000	100,000
Policy	MLP	MLP
Learning rate	0.0003	0.0003
Batch size	256	256
Discount factor (γ)	0.99	0.99
Buffer size	100,000	-
GAE coefficient	-	0.98

**Table 2:** The evaluation results for the agents in the untrained condition for 40 episodes.

Algorithm	Mean Reward	Standard Deviation	Success	Accuracy
TRPO	38.40	7.31	39	97.5%
SAC	39.02	0.71	40	100%

## Data Availability

All data for this study have been experimentally generated and have been included in this paper.

## Abbreviations

The following abbreviations are used in this manuscript:

MLP	Multi-layer perceptron
GAE	Generalized Advantage Estimation
RL	Reinforcement learning
DRL	Deep reinforcement learning
SAC	Soft actor-critic
TRPO	Trust Region Policy Optimization
